# Electroconvulsive therapy use in psychiatric hospitalizations - a nationwide descriptive study

**DOI:** 10.1192/j.eurpsy.2021.1307

**Published:** 2021-08-13

**Authors:** P. Mota, M. Gonçalves-Pinho, S. Macedo, J.P. Ribeiro, A. Freitas, J. Mota

**Affiliations:** 1 Departamento De Psiquiatria E Saúde Mental, Centro Hospitalar do Tâmega e Sousa, Guilhufe, Portugal; 2 Department Of Psychiatry And Mental Health, Centro Hospitalar do Tâmega e Sousa, Penafiel, Portugal, Penafiel, Portugal; 3 2d4h, Center for Health Technology and Services Research (CINTESIS), Porto, Portugal; 4 Psiquiatria, Hospital de Magalhães Lemos, Porto, Portugal

**Keywords:** Electroconvulsive therapy, Mental Health Data, Neuromodulation

## Abstract

**Introduction:**

Despite being one of the oldest treatments in the field of Psychiatry, Electroconvulsive therapy (ECT) is used worldwide for various severe and treatment-resistant psychiatric disorders, establishing itself as one of the fastest and most effective treatments.

**Objectives:**

The primary aim of this study was to describe a nationwide epidemiological perspective of the use of ECT in hospitalized psychiatric patients. The secondary aims were to characterize clinical and sociodemographic trends of hospitalized patients who needed ECT.

**Methods:**

A retrospective-observational study was conducted using an administrative database which gathered all hospitalizations registered in Portuguese public hospitals from 2008 to 2015. We selected all hospitalizations with a procedure code 94.27 - Other electroshock therapy defined by the International Classification of Diseases version-9, Clinical Modification.The variables included in the study were birth date, sex, residence address, primary and secondary diagnoses, admission date, discharge date, length of stay (LoS), discharge status from each single hospitalization episode.

**Results:**

There were a total of 879 hospitalizations with ECT during the 8-year period of the study. Most of the hospitalizations occurred in female patients (53.4 vs 46.6%), belonging to the age group of 51-70 years old, with a mean age of 50.5 years old. The median LoS was 43.0 days with an IQR of 27.0-68.0 days. The specific primary diagnosis most frequent in all hospitalizations was Major depressive disorder, recurrent episode representing 19.6% of all ECT related hospitalizations.

**Conclusions:**

In Portugal most of the patients who received ECT were women above middle age, and depressive disorders were the most common indication.

